# Treatment of Congenital Syphilis in Infants

**Published:** 1875-12

**Authors:** 


### TREATMENT OF CONGENITAL SYPHILIS IN INFANTS.

Sir John Rose Cormack, in an article in the *Medieal Times and Gazette*, gives his treatment as follows:—

In every case a persistent mild mercurial treatment is essential, alternated or supplemented by the administration of small doses of a ferruginous phosphate of lime and cod-liver oil. The preparations of mercury which I generally employ in infantile syphilis are calomel and hydrargyrum cum cretâ. I prefer the former, not because I believe in its superiority, but because I have generally obtained excellent results from its use. It requires, as a rule, to be given together with cretâ preparata to prevent its acting too much as a purgative or intestinal irritant. This was the practice successfully pursued by the late Dr. William Campbell, of Edinburgh. He was in the habit of commencing with doses of a quarter of a grain of calomel and two grains of cretâ preparata once daily for the first ten days. He afterwards progressively increased the calomel to a quarter of a grain twice each day. Of course the age, strength, and general state of the patient must, in each case, determine the amount and frequency of the dose. An infant six weeks old will generally bear well Dr. William Campbell's doses. It sometimes happens that the infant's irritability of stomach is so great as hardly to bear any calomel. In such cases, one of two other simple plans may be tried, viz., the administration of a solution of the bichloride of mercury, or swathing the thighs every alternate eight hours in flannel bands smeared with a mild mercurial ointment. I usually prescribe a solution of half a grain of the bichloride in three ounces of distilled water and one ounce of syrup. Of this solution from one to two teaspoonfuls may be taken every six, eight, or twelve hours, according to the patient and the symptoms. When mercurial swathing of the thighs is adopted, the proportion of mercury in the ointment ought to be moderate, say from one to four drachms of the unguentum hydrargyri of the British Pharmacopœia, to an ounce of lard. Two or three drops of the British Pharmacopœia syrup of the iodide of iron in water, three times in the twenty-four hours, is an admirable sequel to, or occassional change from the mercurial course, which ought to be continued, if possible, for a month, then intermitted for a week or two, and resumed, with occasional short breaks, up to the period of dentition. This is of the utmost importance, for relapses are common, and remissions, to the great detriment of the patient, are apt to be mistaken for cures. Rhubarb, with carbonate of soda, when the bowels are confined, will be found a suitable laxative. The preparation of sulphur called compound power of liquorice in the Berlin Pharmacopœia is also an excellent medicine in such circumstances. When purging or tendency to diarrhœa exists, prepared chalk will generally be found an efficient remedy; but it may be necessary to combine that medicine in a fluid mixture with a very small quantity of the British Pharmacopœia extract of logwood. The calomel sometimes suddenly induces severe and rapidly exhausting catharsis, which has to be promptly checked.

Tepid ablutions and warm baths are very useful; but the latter requires care and judgement, both in prescribing and administering, particularly when the patient is weak. Ulcerated surfaces, cracks, chaps, and cuticular exfoliations must be treated with soothing and healing topical applications. Black wash and the glycerat of borax are both very suitable. Sometimes, in the same case, and at the same time, I employ both. The black wash suits best for healing ill-conditioned ulcerated surfaces, and the glycerat of borax is useful when the soothing and healing of angry sores and large denuded surfaces is demanded. The glycerat possesses the great advantage of keeping the skin in a moist state, and of so preventing painful cracks. The soft, spongy ulcerations often met with around the anus and near the vulva, may be very efficiently dressed with the glycerat of borax, to which there has been added a small quantity of Morson's creasote. The proportions I prescribe are from ten to fifteen drops of creasote to an ounce of glycerat.—*The Medical and Surgical Reporter.*

